# Post-Occupancy Evaluation’s (POE) Applications for Improving Indoor Environment Quality (IEQ)

**DOI:** 10.3390/toxics10100626

**Published:** 2022-10-20

**Authors:** Francesco Lolli, Samuele Marinello, Antonio Maria Coruzzolo, Maria Angela Butturi

**Affiliations:** Department of Sciences and Methods for Engineering, University of Modena and Reggio Emilia, Via Amendola 2, 42122 Reggio Emilia, Italy

**Keywords:** post-occupancy evaluation, building performance, indoor environment quality, occupants’ comfort, literature analysis

## Abstract

To improve buildings and their characteristics, the feedback provided directly by users is generally fundamental in order to be able to adapt the technical and structural functions to the well-being of users. The post-occupancy evaluation (POE) fits perfectly into this context. The POE, through qualitative and quantitative information on the interior environment, makes it possible to identify the differences between the performances modeled in the design phase and the real performances experienced by the occupants. This review of 234 articles, published between 2006 and 2022, aims to analyze and compare the recent literature on the application of the POE methodology. The aim was to provide both a qualitative and quantitative assessment of the main factors that comprise the indoor environmental quality (IEQ). The study highlighted the factors that comprise the quality of the indoor environment, as well as the variables that are usually analyzed to describe the well-being of the occupants. The results suggested which are the most common approaches in carrying out POE studies and will identify the factors that most influence the determination of the good quality of an indoor environment.

## 1. Introduction

People spend a substantial proportion of their time in confined spaces. Approximately 90% of the day is spent at home, work or school, and in traveling [1], so much so that the definition of the “indoor generation” is spreading. In addition, with the advent of the COVID-19 pandemic, the entire world’s population has been forced to stay in confined spaces (especially at home) for long periods of time, which has provided the opportunity to rethink the design and operation of buildings to make them more liveable and efficient in relation to the needs of their users [2].

To improve buildings and their characteristics, the feedback provided directly by users is fundamental information to enable the technical and structural functions to be adapted to the well-being of users [3]. The post-occupancy evaluation (POE), which was introduced in the United States in the 1960s and then disseminated globally, fits perfectly into this context [4,5,6]. This assessment consists of a process of analyzing the characteristics and performance of buildings, carried out with particular attention to the perspective of the inhabitant/user of the building. The concept of the POE is that by asking users about their needs and experiences in the built environment, better spaces can be designed and used [7,8]. By building performance, we mean the behavior of the building system, as a whole, when used by the end user, according to their needs [8]. By allowing a “post-dwelling” evaluation, following the actual use of the spaces by the final recipients of the building, the POE makes it possible to identify any discrepancies between the performances modeled in the design phase and those experienced by the occupants. This reveals whether this discrepancy is due to failures in the building design, construction, management or misuse, and it also identifies improvements that can be made. In this way, this approach combines the information collected through the monitoring of the structural and physical parameters that characterize the living spaces and the qualitative and quantitative indications collected through questionnaires, interviews and visits inside the buildings, directly involving the users [9]. All this makes the POE a useful tool for continuous improvement that is capable of providing useful information to all the actors involved in the life cycle of a building [10,11] and that is applicable to any type of building [12].

According to the authors of [13], the POE evaluates the performance of the analyzed environments according to three main types of aspects: functional, technical and behavioral. Functional performance elements relate to the functionality and level of efficiency of a building’s features, including accessibility, adequacy of spaces and facilities, and services, etc. Behavioral performance concerns the interaction between occupant activities and the physical environment provided. Finally, the elements of the technical performance—such as hygiene and the quality of the indoor environment—represent the factors that influence the comfort, health and productivity of the occupants.

The benefits that the POE guarantees, in the short, medium and long term, are as follows [5]:

- Short-term benefits include obtaining feedback from users about problems in buildings and in identifying solutions.
- Medium-term benefits include the feed-forward of the positive and negative lessons learned into the next building cycle.
- The long-term benefits are aimed at creating databases and at updating, upgrading and generating planning and design protocols and paradigms.

The concept of the POE has evolved considerably over time, adapting to different contexts and applying increasingly complex tools for the collection, processing and combination of qualitative and quantitative data. Therefore, the methodologies for POE are different and there are many ways to conduct it, which characterizes the great flexibility of this approach [14].

As people spend most of their time indoors, indoor environmental quality (IEQ) is one of the priority factors influencing the physiological and psychological health of occupants and results in changes in their habits, well-being, and their physical and cognitive productivity [15,16]. The National Institute for Occupational Safety and Health (NIOSH) describes the IEQ as the quality of a building’s environment, related to the health of occupants within it. IEQ encompasses the conditions inside a building (air quality, lighting, thermal conditions and ergonomics) and their effects on the occupants or residents.

As indicated in [17], parameters such as thermal, acoustic, light and air quality could—also taking into account individual factors (age, sex, etc.) [18]—strongly influence well-being and health, while also playing a role in the performance of the building, such as its energy consumption. Demonstrating the direct relationship between the IEQ parameters and occupants’ comfort has been the goal of many studies: [16,19,20,21,22,23]. For this reason, the POE method is an ideal approach to analyze the interaction between the factors that make up the IEQ and the users of buildings.

In [24], articles that were published between 2000 and 2015 and that identified and classified many indicators to measure the IEQ dimension within the POE’s applications were analyzed. Using POE, each factor was analyzed from the following two aspects: (i) the values of this particular parameter in the environment were analyzed, and (ii) the perception that users have of this particular parameter was assessed [25].

The scientific literature appears to be very rich in articles that analyze the application of the POE approach to different reference contexts, demonstrating its effectiveness as a study tool and as a support for improving planning, management and behavior in a confined environment, both domestic and economic/productive/services.

Several reviews were also conducted, as summarized in Table 1, each focused on particular aspects assessed by the POE or on specific case studies. In [26] the literature on POE, with a particular focus on the origins, theories, benefits and approaches that make up POE, were reviewed by the authors. Similarly, in [27] the authors conducted a critical and exhaustive review of 146 POE projects since 2010 in order to obtain both a qualitative and quantitative benchmark on this issue. The review in [28] critically examined recent case studies of green building certification systems, such as Leadership in Energy and Environmental Design (LEED), Building Research Establishment Environmental Assessment Method (BREEAM), Green Mark and Green Star. In [29], an analysis of the POE tools to identify the methods applied for the evaluation and the metrics used to measure occupant satisfaction was conducted by the authors. The authors of [30] presented the state of the art on the links between IEQs and the well-being and comfort of occupants, with a particular focus on commercial and office buildings. In particular, the literature has analyzed indoor air quality, sick building syndrome, thermal comfort, acoustic comfort and visual comfort, with the aim of providing indications on some primary parameters that characterize the IEQ. The authors of [17] presented a review of the literature about indoor environmental quality (IEQ) and occupant comfort, identifying the most studied parameters. Similarly, review studies [24,31] identify the factors that distinguish each characteristic aspect of IEQs. In [15], the authors, in assessing the health and satisfaction of occupants in green buildings, also analyzed the design, aesthetics and ergonomics of buildings, which characterize the IEQ.

Considering previous work that has examined POE and the various perspectives for future development that have been described, the purpose of this article was to review and compare the recent literature on the application of POE methodology to provide both a qualitative and a quantitative assessment of the main factors that make up the IEQ. Therefore, the specific focus of this review was the application of one/several/all of IEQ’s variables in the POE methodology, by considering the papers that describe the integration of the IEQ into the POE evaluation.

The contribution to the related literature that this article intends to offer is an analysis of the methods used internationally in POE methodology and of the determining factors in defining the IEQ. In addition, in the discussion some limitations of the current literature are identified to guide future research on this theme.

This paper is organized as follows: Section 2 defines the methodological protocol applied in this study to collect, select and analyze the scientific articles. Section 3 presents the results of the literature review from both a descriptive and an analytical point of view. Section 4 then summarizes the main issues and draws conclusions.

## 2. Materials and Methods

To make the literature review more effective, an appropriate approach to the collection and analysis of scientific documents was necessary. All the papers that analyze the concept of indoor environmental quality in the context of the use of the POE approach were considered.

**Figure 1:**
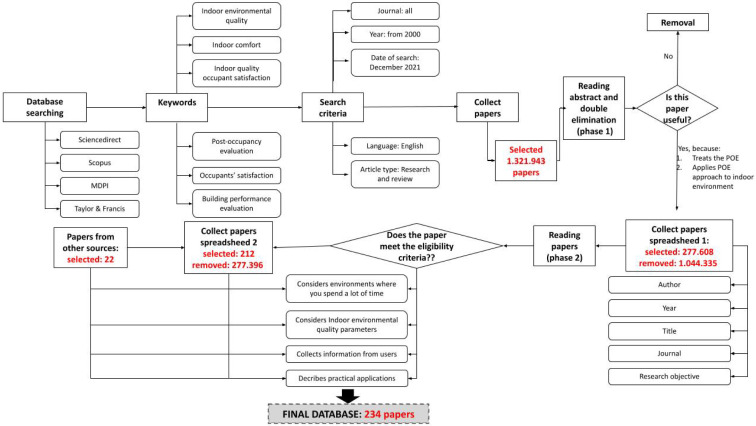
Research and analysis protocol.

Therefore, the search and analysis protocol, shown in Figure 1, was applied. The bibliographic search was conducted using the Scopus, ScienceDirect, Taylor & Francis and MDPI databases, with a search restricted by year (since 2006), type of publication (search and review) and the English language. No restrictions were imposed on the journal. These choices are intended to foster the ability of research to capture the latest developments in the practice of POEs, despite the fact that the POE method originated in the 1960s and, therefore, there are also publications dating back to that time. Several keywords were used, divided into two groups, which were referred to as “Group A” (post-occupancy evaluation, occupants’ satisfaction and building performance evaluation) and “Group B” (indoor quality occupant satisfaction, indoor comfort and indoor environmental quality). The first group focused their research on the primary object of the study (POEs), while the second group specialized in the IEQ aspect. Each keyword in Group A was searched, coupled with the others in Group B using the “*” wildcard character, which was employed for multi-word searches. The list of combined keywords were used to search the selected databases.

The collected articles were analyzed by applying a scalar approach to the evaluation of their content, from a preliminary level to a more in-depth analysis of the content. The selection eliminated duplicates and analyzed the abstracts of the articles to assess their conformity to the objective of this article. The remaining articles were classified according to the author’s name, the year of publication, the title of the paper and the keywords used and, finally, they were evaluated by applying eligibility criteria.

A full-text evaluation was then carried out. It was only with this last step that the selection was completed. Finally, by browsing other known references and tracing the references in the selected documents (backward snowballing), other contributions were identified. This process resulted in a total of 234 papers.

The critical analysis of the selected articles was carried out through a structured approach to extract the descriptive elements from the scientific literature and the parameters on which to conduct the critical analysis. The descriptive analysis provided a general overview of the material collected, highlighting three key aspects that framed the reference bibliography: year of publication, journal and field of study. The critical analysis assessed the content of the articles in relation to the following: (i) the objective of the study, (ii) the type and number of buildings analyzed by each author, (iii) the modalities of involvement of building users and the number of responses collected, (iv) elements considered, and (v) type of factors that make up the IEQ dimension considered, and any physical parameters measured.

## 3. Results and Discussions

The results of the descriptive and critical analyses are reported below. A total of 234 articles were collected.

### 3.1. Descriptive Analysis

**Figure 2:**
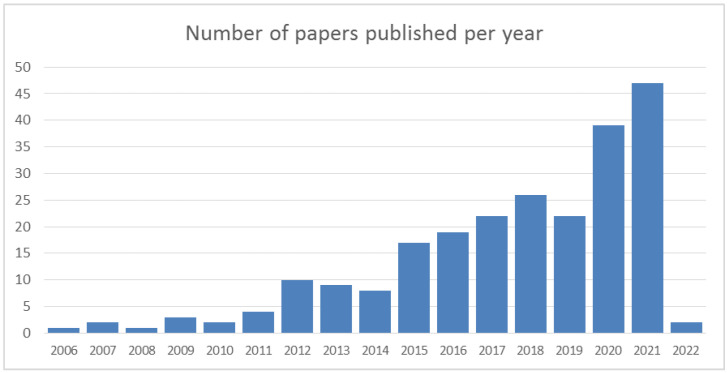
Number of articles per year of publication.

The study period (2006–2022) was covered by articles dealing with the issue of POE methodology (Figure 2). The period from 2015 was particularly rich in scientific contributions, which represented more than 83% of the articles selected in this journal. The years 2020 and 2021 were the most represented years, with 39 and 47 articles published, respectively.

The journal that had the largest number of publications was *Building and Environment* (approximately 25% of the articles analyzed were from here). This underlined that this international journal is strongly oriented towards the themes of the analysis of buildings and the interaction between people and the surrounding environment. Approximately 9% of the articles were published by *Energy and Buildings*, 5% by *Buildings* and 3.5% by *Sustainability*. In addition to these, there were 60 other different journals that published at least one relevant article. Some contributions (6.5%) were published in the conference proceedings.

The selected studies focused specifically on the UK and the US, with 10% of the articles each, followed by Australia (9%), China (7%) and Malaysia (6%). In Europe, excluding the UK, there were another 20 papers (approximately 10% of the total analyzed) that were fairly evenly distributed among the Member States. It is interesting to note the high concentration of papers that also came from developing countries (e.g., Nigeria, Ghana, Liberia, Sri Lanka and South Africa), which represented 7% of the papers.

### 3.2. Critical Analysis

The results described in this section relate to the five aspects that made up the methodological protocol reported in the previous section. A subsection has been dedicated to each aspect.

#### 3.2.1. Objective of the Study

This assessment summarizes the end goal that the authors intend to achieve through their case study. The objectives stated by the authors have been classified into the following categories:

- Overall comfort/satisfaction of the occupants.
- Specific aspect of occupant comfort against the factors that make up the IEQ.
- One or more specific factors that make up the IEQ, as follows: indoor air quality, acoustic comfort, thermal comfort and visual/light comfort.
- Building performance.
- Safety requirements.
- Satisfaction–productivity ratio.
- How POE supports building design or maintenance.
- The effect on occupant behavior.
- General description.

Table 2 shows the number of papers describing each objective and the relative frequency in percentage. Most of the authors (approximately 44%) pursued the objective of applying the POE to assess the comfort/satisfaction of the occupants, with respect to the various factors that characterize the functionality of the building. These applications are very heterogeneous, and the POE makes it possible to analyze the technical, functional and psychological factors and variables, thanks to the involvement of the users.

The authors of [36] used the POE to determine the level of user satisfaction with university shopping mall facilities by evaluating the satisfaction of the occupants with respect to the following five categories of factors: building performance, safety, proximity and accessibility, planning and layout of space, and mall services. Furthermore, in the school environment, Ahmed et al. (2021) [14] assessed several school renovation projects in the UK, noting the need for greater control of the internal environment, thus contradicting the current trend towards automated “intelligent systems” approaches. The authors of [37] applied POE to assess occupant satisfaction with school facilities, by applying a novel approach to assess the key technical, functional and behavioral elements of the performance of schools in Saudi Arabia. Kim et al. (2015) [38] assessed occupant comfort and satisfaction in green healthcare environments, using a case study to investigate the real-world factors that influence occupant comfort and satisfaction with the healthcare provided, by comparing the perceptions of the healthcare staff in green hospitals with those in conventional hospitals. The results obtained highlighted how green features can have positive effects on healthcare staff, suggesting them as relevant elements for these structures.

The authors of [39] showed how the use of POE in the school environment can also be applied as a tool for collecting technical and functional indications for the redesign of the school. In [40], the level of satisfaction with the level of technology (television monitors, DVD and video cassette recorders, overhead projectors and slide projectors, and video presenters), the thermal characteristics of the environments and the level of cleanliness of an establishment of university education in the Midwest, United States, were analyzed by the authors. The authors of [41] analyzed schools in Iceland that combine open and confined spaces, designed for multiple pedagogical approaches and multiple uses, highlighting the design strengths and weaknesses. In [42], college dormitories across POEs based on the socio-technical systems approach were analyzed by the authors to identify factors that contribute to student satisfaction with their residence. The authors of [43] identified and prioritized the social impacts of high-rise residential buildings in Tehran (Iran). Anti-social behavior, a lack of social cohesion and a lack of social contact with neighbors were the factors identified as the most significant. The authors of [44] assessed the liveability and comfort of social housing in Liberia from the perspective of the residents.

The IEQ is the second most studied element in the literature (approximately 36% of the articles analyzed in this review studied this). It is important to point out that IEQ factors are often items that are assessed by the POE, along with other technical and functional items, and which, in this review, have been listed in the previous category, called “occupant comfort/satisfaction”. In [45,46,47], occupant satisfaction with IEQ was analyzed by the authors against buildings with and without LEED or BREEAM certifications. The studies analyzed office buildings in the UK in terms of air quality, thermal comfort, ventilation, building characteristics and light. The authors of [48] applied a protocol for POEs in schools to improve the indoor environmental quality and energy efficiency, assessing the following aspects: visual, air, thermal, acoustic and spatial quality. In [49], they presented the results of a study conducted in 32 schools in Manitoba (Canada), in which they used the POE to assess the satisfaction of the IEQ for occupants and to analyze its use as a measure of the psychological, social and physical well-being of the teachers. The results showed that ventilation and thermal comfort were the most significant measures. Similarly, the authors of [50] assessed the job satisfaction and self-reported productivity of university employees, with respect to ventilation and thermal comfort, acoustics, privacy, and lighting. In [51,52], they developed an integrated occupant psychological response score, based on the activities of 22 experimental participants and changes in the IEQ conditions. The authors of [53,54] analyzed the context of commercial buildings in Australia, evaluating the ways to improve occupant satisfaction through better indoor conditions. In [55], the effect of library interiors on occupant satisfaction and performance was assessed by the authors.

However, some authors analyzed a specific aspect that characterizes the IEQ. For example, the authors of [56] examined the thermal comfort of occupants during the dry season in low- and middle-income residential buildings in Abuja, Nigeria. In the study, post-occupancy surveys were used to assess the adaptation of buildings and residents’ adaptation to the thermal environment. In [57,58], they assessed thermal comfort in schools and historical museum buildings in Spain. The evaluation of this parameter made it possible to highlight the need to explore the possibilities of lowering indoor temperatures, in particular passively (fabric, shade, insulation, etc.), taking into account the need to avoid or reduce the need for air conditioning to make buildings energy efficient for lower–middle income groups. In [18], the authors monitored the air quality of a renovated building and assessed the impact of sick building syndrome (SBS) on the occupants. The results of the study showed a direct relationship between a high airborne mold, TVOC and the negative health perception of the building’s staff. Similarly, the study in [59] features case studies of offices in Denmark. In [60,61], the authors deal with noise analysis in residential buildings. Finally, the evaluation of visual discomfort as a characterizing element of the IEQ is described in [62,63,64,65].

Less common, were studies that had other objectives. In [66,67,68,69,70], the authors presented some studies where the POE was used to analyze the performance of buildings from a functional and technical point of view (approximately 7% of the papers analyzed). Entrance space in residential apartments, energy retrofits and consumption, water consumption, and feedback from facility managers were some of the factors that were evaluated.

Some authors (approximately 7%) applied the POE to identify the priority aspects to plan the necessary interventions on the buildings and the obstacles inherent in these works. Some of the studies that were oriented in this direction were [71,72,73,74,75].

Other studies applied the POE to facilitate improved occupant behavior (2%), as described in [76,77,78,79]. Another 2% of the authors used the POE as a tool to support building maintenance actions, [80,81,82,83]. The latest objectives analyzed, which were each investigated by approximately 1% of the authors, provided for an improvement in the satisfaction–productivity ratio ([20,45]) and assessed the safety requirements ([84,85]).

#### 3.2.2. Buildings Analyzed

POE is a very versatile tool that can be applied to different contexts. This aspect reflects the different types of buildings discussed by the authors and the buildings’ state of use (new, renovated, already inhabited/used buildings, Table 3). Offices represented the largest number of studies available (approximately 36% of the total analyzed), in particular, to improve the satisfaction and productivity of their occupants. The authors of [86] analyzed 398 offices in Malaysia, with specific reference to the ability of the heating, ventilation and air conditioning (HVAC) systems to provide a comfortable working environment. Assessing the sustainability of 140 sites, in [87] the authors applied 45 factors that were representative of operational, environmental, personal control and satisfaction aspects, and collected user perception scores via the POE tool.

More recent, were the studies described in [88,89,90,91]. The authors of [88] analyzed indoor environmental quality (IEQ), combined with environmental measures, to examine the satisfaction of the existing occupants. In [90], the authors analyzed which aspects supported the development and improvement of their performance towards the objectives of green building certification. A series of 2657 certified office building records were assessed. Finally, the authors of [91] analyzed 36,671 post-occupancy evaluation responses to determine which indoor environmental quality parameters best predicted the overall workspace rating.

The second main type of building analyzed were educational environments (at different levels, from small schools to universities). Approximately 29% of the authors analyzed this category. Again, POE ensures the best possible conditions for its users. The authors of [92] compared a number of quantitative and qualitative aspects of use in a sample of 10 conventional schools, 20 energy-retrofitted schools and three green schools in Toronto. In [93], the authors applied the POE as a preventive tool to assess which factors could influence the behavior in 47 naturally ventilated classrooms in southern Europe, in relation to climate change and its effect on the average temperatures and duration. The authors of [94] analyzed the IEQ and the influence of buildings characteristics in 26 schools in Spain.

The last predominant category was composed of residential and domestic buildings, and was analyzed by approximately 21% of the selected authors. The application of the POE aimed to support the improvement of the comfort of the residents as well as the sustainable performance of the installations and their operation. In [95], the authors measured indoor climate conditions in high-performance residential buildings in Norway. The authors of [53] evaluated the living experiences of the residents in 199 apartments, which made up a modular multi-residential development in central Melbourne. The authors of [96] studied occupant satisfaction and awareness through the performance of 158 green homes in the UK. In [97], the authors applied POE to a social housing complex, consisting of 400 apartments, to investigate the causes and possible solutions for high humidity levels. Yang et al. (2020) [98] studied IAQ, energy and occupant satisfaction and behavior in 650 energy-efficient homes in French-speaking Switzerland. The authors of [99] analyzed 361 LEED-certified and non-certified apartments using social media data.

The other building types (which, together, accounted for 15% of the studies analyzed) applied POEs to a variety of structures. For instance, in [7,100,101] the authors applied POE to existing hotel and hostel buildings to assess their functionality against the current needs, and also to be able to adapt to new changes in use. The authors of [102,103,104] applied POEs to student residences. The authors of [105,106,107] analyzed historical and cultural environments, with applications to museum, library and other historical buildings. Sports facilities were the focus of the analysis conducted in [23], with an experimental application at a gymnasium in eastern Ontario, Canada. Two studies, [108,109], analyzed health facilities and hospitals. Finally, to assess religious structures, in [110] they conducted a study of mosques in Saudi Arabia.

It is evident that the most investigated buildings were those already inhabited/used, with minimal differences between the different types of buildings (e.g., hotels/hostels/B&Bs were 100% already used, while residential buildings were 89% already inhabited, 3% refurbished and 8% new).

#### 3.2.3. Collection of Qualitative Information

Another aspect characterizing the application of the POE concerns the means of engaging the users of the environments analyzed and the tools implemented to collect their responses. As stated in [13], questionnaires in POE studies are the most important part of any building evaluation study, because buildings that do not meet the requirements of their users cannot be classified as performing well, even if their physical measurements are satisfactory.

Table 4 shows the means of involving users that were described by the authors. The use of digital solutions (internet, social media, email, etc.) are currently the most widespread tools, applied by 73% of authors. In [111], to assess indoor air quality and occupant satisfaction in five mechanically ventilated and four naturally ventilated open-plan office buildings, the authors distributed a POE questionnaire via a web-based approach. In [112], the authors, in their assessment of green and conventional university buildings, distributed the questionnaire via email, receiving 319 responses. In [113], the authors sent the questionnaire via email in order to evaluate the IEQ by comparing a building classified as Green Mark Platinum, with a building not classified as Green Mark. In [114], the authors used an online survey questionnaire, distributed via email, through the facilities manager, to the occupants of the three buildings selected for the case study. The questionnaire assessed their environmental awareness, perceptions, and the perceived ease or difficulty of pro-environment behaviors. Out of the 883 potential survey responses, 106 responses were collected. In [115], to assess the energy certification of the building with regard to user satisfaction with the indoor environment, the authors collected 277 responses via an online survey, and 269 via a point-in-time survey.

The studies analyzed in this paper, describe a very different number of samples (in terms of the number of responses received). For example, in [74] the authors collected 14 questionnaires to assess changes in household practices before and after employment. In [116], in the evaluation of centralized and decentralized ventilation systems in residential buildings in Luxembourg, the authors collected 16 questionnaires. To analyze thermal comfort and occupant satisfaction in the office, in [117] they collected 20 questionnaires. In [118]—a building performance assessment for sustainable agri-food production—the authors collected 24 questionnaires.

Other authors, on the other hand, managed to collect a very high number of responses, such as in the following: in a case study on eco-certified buildings, [46], 11,243 were collected; the authors of [40] obtained 5173 responses on class spaces; the authors of [119] collected 5756 responses, with an indication of overall satisfaction in Swedish households; and the authors of [120] collected 4086 responses on three-star certified office buildings.

On average, across all the articles analyzed, the number of interviews was 588, highlighting the great potential of this approach, compared to the average number of samples collected by the other methods, shown in Table 4.

Similarly, the response rate (i.e., the number of responses received, compared to the number of requests sent) was highly variable, ranging from 7% to 94%. The average value was 52% [121], with 85 samples collected describing a 7% response rate. Two studies, [77,122], reported a similar percentage, but with a much higher number of samples (278 and 729, respectively). The authors of [123] achieved an 85% response rate, with 100 samples; the authors of [124] achieved 88%, with 46 samples; and the authors of [50] achieved 92%, with 65 samples. The highest percentage (94%) was reported in [18], in which the authors obtained 94 samples.

The main drawback of this approach is the difficulty of collecting in-depth information and the need for a simple, intuitive and fast questionnaire-response-time structure.

Conversely, personal interviews provide a better understanding of the topics of the interview but take a lot of time to organize and manage. However, this solution seems to be quite widespread (21% of authors, such as [125,126,127]). Furthermore, in this case, some authors managed to collect a limited number of responses (e.g., [73,109,128], who worked with 8, 16 and 22 responses, respectively). In [129], they obtained 796 responses, in [39] they obtained 577, while in [55] they obtained 556.

From a response-frequency perspective, ensuring direct contact with building users was important—at least 20% of those contacted provided an answer [130]. The average value was 47% and the maximum value was 75%, as reported in by the authors in [63].

On the other hand, the number of paper questionnaires distributed to users (5% of authors) and the number of telephone interviews conducted (1%) in the studies evaluated in this review were marginal. The first tool, as described by the authors in [131,132,133], was applied to prefabricated timber houses in the UK, government buildings in the Kingdom of Bahrain, and universities in Cairo (Egypt), respectively. The second, on the other hand, was described by the authors in [134], in which it was used to understand how facilities’ managers reacted to user feedback and its impact on users’ post-feedback behaviors.

Another relevant aspect in the collection of questionnaires was represented by the number of times the responses were collected. In most cases (79%), the authors did not replicate the sampling, doing it only once. In some cases, user opinions were collected multiple times.

#### 3.2.4. Elements Considered

The articles collected were also assessed according to the type of elements analyzed by each study. In particular, according to the authors of [13], the different performance elements considered in post-housing assessments can be divided into the following:

- Functional performance elements, which refer to the level of functionality and efficiency of elements in buildings, including accessibility, the suitability of spaces and structures, and services, etc.
- Behavioral performance elements, which relate to the interaction between occupant activities and the physical environment provided.
- Technical performance elements, such as hygiene and the quality of the indoor environment, and all factors that influence the comfort, health and productivity of the occupants.

Functional performance elements were investigated by 177 papers, which represented 82% in terms of frequency. Behavioral performance elements and technical performance elements were moderately less investigated: 77% and 78%, respectively.

Often these three aspects were assessed in the same study, as described by the authors in [135,136,137,138,139,140,141,142,143]. In other studies, only one or two aspects were considered at one time ([110,144,145]).

#### 3.2.5. IEQ Parameters

The interaction between the IEQ and occupant satisfaction is very complex. Due to the numerous studies available (e.g., [17,22,46,50,106,146]), it has been shown that the IEQ has a direct short- and long-term effect on the comfort, health and productivity of the occupants of buildings. Therefore, to analyze these aspects, the POE also contributes to the evaluation of all the possible factors [31]. As reported in [25], the factors that influenced IEQ and occupant satisfaction can be divided into physical factors (thermal comfort, indoor air quality, lighting and acoustic environment), which can be assessed by corresponding measurable parameters and non-physical factors (layout of space, privacy, cleanliness, facilities, and the view from the building) that are difficult to measure with tools.

Most of the authors (88%) considered the IEQ parameters in the case studies described as supporting elements for understanding occupant satisfaction and for planning possible improvements. In some cases (21%), the IEQ factors were not taken into account.

The authors of [147] compared the expected and actual energy performance of non-residential buildings and applied the POE to produce more accurate energy performance models. The authors of [81] used the POE to prioritize maintenance work to achieve the maximum occupant satisfaction. The authors of [68] aimed to improve the accuracy of buildings’ energy simulation, through the evaluation of occupant behaviors. The authors of [67] evaluated the functional performance of entrance spaces in apartments in the Kurdistan region of Iraq.

With respect to the physical parameters, the analyzed authors described whether or not the evaluation of the POE was completed by in situ measurements, or whether the evaluation was carried out solely by qualitative assessments and judgments, provided by the users of the spaces investigated. In this case, there was a greater balance: 43% expected physical measurements, while 57% of the authors only made qualitative assessments. Some case studies that complemented the POE surveys with field measurements were as follows: [148], alongside the questionnaires, the authors also measured the temperature, relative humidity, noise, light, and CO2 concentrations in the case study of Universiti Teknologi PETRONAS’ new academic complex; the authors of [149] measured the brightness of highly glazed modern buildings, which require solar protection to ensure the visual and thermal comfort of the occupants; the authors of [150] detected the temperature and relative humidity trends in houses in the hot and humid tropical climate of Darwin; the authors of [151] measured the brightness in collective housing in Algeria; the authors of [152] measured different physical parameters (temperature, relative humidity, noise, light and CO2 concentrations) for a comparative study on green and conventional malls in Beijing, China; the authors of [61] focused on noise measurements to improve the acoustic performance of residential buildings in Turkey.

Conversely, some authors only carried out qualitative evaluations, as follows: the authors of [135] analyzed the relationship between defects and occupant satisfaction and loyalty in build-then-sell houses; the authors of [86] assessed how maintenance features might affect occupant satisfaction; the authors of [71] analyzed the demand for space in the common areas of student residences in Iran; the authors of [153] applied the POE to understand how occupants perceive wood in built environments; the authors of [129] analyzed how the social dimension of physical space in educational contexts can explain a student’s academic achievement.

Considering only the 157 articles that provided qualitative and/or quantitative assessments of IEQ’s physical parameters, Table 5 reports the frequency of analysis of the four categories proposed in [25]: thermal comfort, indoor air quality, lighting and acoustics. For each variable investigated, the main types of tools used for measurement were also reported, as support elements for the design of measurement campaigns, which are useful for the use of the POE methodology.

All categories were considered by a significant number of authors. The thermohygrometric evaluation of environments was the most considered category (85%), in particular, by measuring the temperature, relative humidity and atmospheric pressure. The authors of [154] described the importance of thermal control for building occupants and facility managers. The authors of [155] described the case study of a LEED gold-rated university building, in which thermal comfort was analyzed as an IEQ factor. In [42], with a case study of university dormitories in China, they described residential satisfaction, which was also linked to thermal comfort. In [118], in evaluating the performance of an agri-food building in Italy, they measured the indoor heat levels in hot and cold seasons. In two studies, [16,156], the authors measured the thermal conditions in green office buildings in Jordan.

In [157], evaluating 20 office buildings, they measured the lighting levels as a factor in visual comfort and workplace productivity. The authors of [158] analyzed the internal conditions of the Arts Tower (Sheffield, UK), also through light measurements. The authors of [159] analyzed the physical quantities of lighting in an open plan office to assess the layout of the environment and the effect on the occupants. The authors of [160] compared green office buildings with different levels of energy consumption intensity, using light measurement as a rating indicator. The authors of [161] analyzed the IEQ of platinum, green-certified office buildings in Malaysia, assessing thermal comfort, indoor air quality, acoustics, lighting, furnishings and cleanliness.

The authors of [162], by monitoring the performance of four social housing units certified to the Code for Sustainable Homes level, measured CO_2_ levels as a representative parameter of indoor air quality. Likewise, the authors of [115] applied CO_2_ measurements as an air quality indicator to assess energy-certified buildings. The authors of [54] also used this indicator to assess the quality of office space at an Australian urban university. In [163], the authors verified occupants’ satisfaction with the indoor environment at work, through the qualitative assessment of indoor air quality. In [164], for the assessment of green office buildings, they measured many air pollutants, such as CO_2_, PM2.5, CO and formaldehyde. The authors of [165], in a zero-carbon building, measured CO_2_ and PM2.5 concentrations.

Finally, noise was analyzed in 62% of the papers collected. The authors of [166] quantified several key factors influencing occupant satisfaction in higher education institutions in the USA and Lebanon, considering acoustic quality as one of them. The authors of [167] analyzed the environments of university buildings in Chongqing, China, through questionnaires and occupant measurements. The authors of [168] analyzed the office plan of the Land Rover/Ben Ainslie Racing (LR/BAR) team’s headquarters in Portsmouth, UK. A comparison was made between the measurements with the occupants’ perception of comfort with respect to the same parameters. The authors of [169] examined occupant satisfaction in three excellent BREEAM-certified buildings at Coventry University, in the UK. Qualitative assessments were carried out on the perceptions of the occupants, evaluating the thermal environment, indoor air quality, as well as the visual and acoustic environment. The authors of [11] analyzed satisfaction in the office buildings of the University of Southampton (UK), by measuring and evaluating the thermal, acoustic and air quality of the indoor environment.

Table 6 shows the relationships between the four categories of the physical parameters that describe the IEQ. Most authors (68) considered, in the same case study, qualitative or quantitative evaluations of parameters relating to all the categories considered (indicated in the graph by “ABCD”). Considering three categories simultaneously, the most frequent combination (12 studies) considered lighting, acoustics and thermal categories (indicated in the graph by “ABC”). Thermal comfort and indoor air quality were the two most studied categories (16 studies). Finally, when a single category was analyzed individually, thermal comfort was the most investigated aspect (17 studies), followed by light, in 10 papers.

## 4. Some Future Work, Suggested by the Literature

Thanks to the information provided by the extensive literature analyzed, some useful elements for future research are reported below, with the aim of supporting studies and providing insights on the use of the POE methodology to improve the quality of indoor environments and to ensure greater well-being for people who live in, work in and use these spaces, as follows:

- Apply the POE for cost–benefit analyses.
- Adopt larger samples to draw conclusions with greater statistical power.
- Apply multi-criteria assessment approaches to better represent the occupants’ satisfaction.
- Due to the cultural differences, the results and recommended solutions of the POE’s applications might not be necessarily generalizable to other contexts. Therefore, models that allow the transfer of results are a relevant requirement.
- Conducting a study focusing on individual differences in the sensitivity to the IEQ would be helpful to address the generalization limitation.
- Develop and test exemplary standards that provide market companies, professionals and decision makers with the most applicable procedures at the same time.
- Future studies using occupant voting systems need to be conducted over a long period (at least 30 days), or that, at least, introduce interventions to create high variations in indoor conditions and occupants’ voting patterns.
- Greater diffusion of the use of the POE to include repair, maintenance and refurbishment projects.
- Develop interfaces and communication supports that allow the results of the POE to be applied in signaling the elements of the building that are the source of the problem to the occupants and to the designers employed to find solutions.
- Use the POE on the diverse responses of occupants (e.g., psychological, cognitive, physiological and emotional) for different project solutions through a virtual environment.
- Conduct post-COVID-19 assessments through POE application.

## 5. Conclusions

We have presented a critical analysis of the main results concerning the application of the POE in the evaluation of the IEQ of indoor environments, as a support for the improvement of internal comfort and user responses in terms of, for example, productivity in the field—in the case of indoor environments—and work or satisfaction—in the case of the inhabitants or customers of a public body.

The vast bibliography available on this subject has been analyzed according to specific aspects, as follows: year of publication; journal; case study; country; the purpose of the study; the type and number of the buildings analyzed by each author; the terms of involvement of the buildings’ users; the number of responses collected; the elements considered; the type of factors that make up the IEQ dimension considered; any physical parameters measured that provide a clear picture of the current literature to researchers and practitioners; and some proposals for further research agendas, which could address the current literature’s limitations.

The conclusions of the study, including some limitations, are as follows:

- There are many scientific studies describing case studies on the application of POE. The number of these studies has grown significantly, especially since 2015. The last years of research (especially 2020 and 2021) have been particularly attentive to this issue. This highlights the importance of this tool, which is still extremely widespread, despite being available for more than 60 years.
- Scientific research, from a geographical point of view, is well distributed among the different regions of the world, including many developing countries (e.g., Nigeria, Ghana, Liberia, Sri Lanka and South Africa).
- The use of the POE pursues the main objective of evaluating occupant satisfaction and IEQ comfort. It has also demonstrated its ability to support technical actions to improve the performance of indoor environments, e.g., for design, maintenance, etc. This testifies to the flexibility of the POE and its ability to meet different needs.
- First and foremost, offices, schools, and private residences are the target audiences for surveys that are conducted through the POE. However, there is no shortage of applications in specific contexts, such as hotels, hospitals, museums and churches. This aspect also enhances its flexibility
- Digital tools for conducting questionnaires have made it possible to obtain a very high number of responses, thus broadening knowledge and increasing the statistical representativeness of the samples analyzed. Traditional approaches (such as personal interviews) are still used, and they provide significant results in terms of the detail and accuracy of the data collected.
- Quantitative measurements are often also associated with qualitative interviews, through the monitoring of specific physical parameters. These assessments generally concern the different categories of indoor parameters, such as thermal comfort, indoor air quality, light and the acoustic environment. Temperature, humidity and atmospheric pressure are parameters that were measured in almost all the case studies that we evaluated, perhaps because of the low cost of the tools required. The variables that best represent the other categories were detected less frequently (but still significantly).

## Figures and Tables

**Table 1:** Review articles related to POE methodology.

Paper	Objective	Consider IEQ	Paper’s Contribution	Gap and Future Developments
Afroz Z. et al., 2020 [28]	POE evaluation applied to certified buildings.	Yes, as part of green building projects.	Collect a large amount of information related to post-employment data collection and analytical approaches prescribed by the certification systems reviewed.	- Discrepancies in data infrastructure and archiving practices. - In-depth policy exploration and strategies suggested by the certification schemes. - Further research efforts in utilizing the data for advanced-level analysis.
Al Horr Y. et al., 2016 [30]	Describe the state of the art about the links between IEQs and occupant well-being and comfort.	Yes, assess the different factors that make up the IEQ.	The relationship between the IEQ and the well-being of the occupants and the relationship of IEQs amongst themselves is quite complex. Green building designs do not automatically guarantee that the building designed will be comfortable and ensure occupant well-being.	- More specific and in-depth reflections on the well-being of the occupants necessary. - Designing a potentially comfortable building is not enough. It is also necessary to monitor the performance of the building and its occupants during its operations.
Aliyu A.A. et al., 2016 [26]	Review previous literature on POE—origins, theories, benefits and approaches used in conducting POE	Not a priority	POE facilitates the detection of construction defects at an early-stage so corrective actions can be implemented as soon as possible.	- Future multidisciplinary research is recommended—deepen the social aspects. - Learn more about how occupants experience buildings. - Examine trends and patterns in building energy data. - Promote the successes of the development of social housing.
Artan D. et al., 2018 [29]	Review metrics used to measure occupant satisfaction, information collected for each parameter and mechanisms adopted to process the data collected.	Yes	The results show that most of the existing tools are not statistically validated as a measurement construct and that there is no consensus on occupant satisfaction measures, as well as on the information that should be collected by the operator/ occupant for each parameter	- Improve the way data are collected and managed.
Brambilla A. and Capolongo S., 2019 [32]	Compare and review recent tools able to assess the built environment of the hospital	Yes	The most recent tools analyzed by the document show a tendency to increase the percentage of indicators related to health rather than sustainability.	- Understand the effectiveness of those tools in practice.
Durosaiye I.O. et al., 2019 [6]	Describe the state the art of POE in the UK building procurement process.	Not a priority.	POE can be used to make important strategic decisions. Facility managers can use information from this POE repository to make strategic decisions.	n.a.
Esfandiari M. et al., 2017 [17]	Analyze IEQ parameters and their relationship to occupant satisfaction.	Yes.	Identify IEQ parameters that have a strong influence on occupant comfort. The thermal, acoustic, light and air quality could strongly influence the comfort and health of people, playing a critical role in the energy consumption of buildings. There is a complicated relationship between IEQ parameters, which makes it difficult for a designer to find a balance between them.	- Simultaneously identify full satisfaction and IEQ parameters.
Fantozzi F. and Rocca M., 2020 [31]	Collect indicators for occupant health and comfort assessment in IEQ assessments.	Yes.	Human health risk assessment and comfort assessment indicators are specified.	- Simultaneously identify full satisfaction and IEQ parameters.
Galasiu A.D. and Veitch J.A., 2006 [33]	Occupant preferences and satisfaction with lighting environment and control systems in daylight offices.	Yes, for daylight.	The paper reveals the limitations in the current knowledge about how people react to daylight and, in particular, how they react to lighting and shading controls. Improving the energy efficiency of commercial buildings’ lighting should include better use of daylight, but this will require the development of control systems.	- Systematically study the lighting conditions created by individuals using manual lighting and shading control systems. - Make systematic comparisons. - Widen the range of light conditions studied. - Study the relationship between discomfort and glare ratios.
Geng Y. et al., 2019 [25]	Review published research on post-occupancy performance of green buildings in terms of energy consumption, IEQ and occupant satisfaction.	Yes, with special attention to green buildings.	The energy performance of green buildings was, on average, better than that of conventional buildings. A significant discrepancy was found between planned and operational power consumption. It was not possible to observe a clear relationship between the actual energy consumption and the level of certification of sustainable construction. Current IEQ conditions of green buildings were not comparable in different countries. Green buildings generally have a higher level of occupant satisfaction than conventional buildings.	- New data collection technologies. - Global performance optimization.
Ilter D.A. et al., 2016 [24]	Collect indicators for assessing occupant satisfaction in IEQ evaluations.	Yes.	Evaluation indicators.	- Identify and solve existing issues that impede occupant satisfaction and guide the design of retrofits in office buildings to maximize building performance and user needs.
Lee J.W. et al., 2020 [34]	Implement a web-based building occupant tracking system that incorporates the new approaches, based on a geographic information system (GIS) tool and open source spatial information.	Yes.	Define a detailed system framework	- Conduct research on IEQ factors. - Analyze occupant satisfaction and display the visualization vertically. - Carry out a case study of the occupants of a real building to propose a direction for statistical analysis with 3D visualization.
Li P. et al., 2018 [27]	Qualitative and quantitative introduction of POE.	Yes.	Emerging research topics related to visualization of POE results, occupant survey database analysis, and occupancy measurement.	- Five directions for future POE development and applications, as follows: from ad hoc to ongoing, from high-level to detailed, owner-/occupant-oriented researcher-oriented, from academia to industry, and from independent to integrated.
Meir I.A. et al., 2009 [5]	Describe POE’s conceptual and methodological context, its interaction with other issues related to sustainable design and its growing “canonization” as a method.	Yes	POE is an important and probably inevitable step to make buildings more sustainable.	n.a.
Mirzaei N. et al., 2020 [15]	Examine the relationship between buildings and health	Yes	Identification of important IEQ factors, including building design, aesthetics and ergonomics, which were less valued in previous research. Occupants of green buildings enjoy higher IEQ, satisfaction and health than occupants of non-green buildings.	- More buildings to accurately assess the indicators cited.
Roberts C.J. et al., 2019 [35]	Analyze POE literature on building operations and performance as a way to holistically map the body of existing knowledge	Yes	A stronger community of practice is needed to ensure a consistent approach to POE.	- Expand current research study and generate broader debate among practitioners and scholars.

**Table 2:** Aim of study.

Objective	Number of Papers	Frequency %
Occupant comfort/satisfaction	90	38%
IEQ comfort	73	31%
Design support	15	6%
Building performance	14	6%
Help with maintenance	5	2%
Improvement in occupant behavior	4	2%
Safety requirements assessment	2	1%
Satisfaction–productivity ratio	2	1%
General description	29	12%
Total	234	100%

**Table 3:** Type of building analyzed.

Type of Building	Number of Papers	Frequency %	State of Use of the Buildings
Office/commercial building/public building	73	31%	New: 7% Restructured: 0% Already used: 93%
Educational institution	59	25%	New: 5% Restructured: 0% Already used: 95%
Residential building	43	17%	New: 8% Restructured: 3% Already used: 89%
Others	8	3%	New: 22% Restructured: 1% Already used: 77%
Hotel/Hostel/B&B	5	2%	New: 0% Restructured: 0% Already used: 100%
Students’ halls of residence	5	2%	New: 0% Restructured: 0% Already used: 100%
Museum/library/historical buildings	5	2%	New: 0% Restructured: 0% Already used: 100%
Fitness building	3	1%	New: 0% Restructured: 0% Already used: 100%
Healthcare	3	1%	New: 0% Restructured: 0% Already used: 100%
Religious structures	1	1%	New: 0% Restructured: 0% Already used: 100%
Not available	29	12%	n.a.
Total	234	100%	

**Table 4:** Type of approach used.

			Number of Responses			Response Rate (%)		
Approach	Number of Papers	Frequency %	Min	Mean	Max	Min	Mean	Max
Online questionnaire	113	48%	14	588	11,243	7	52	94
Personal face-to-face interview	33	14%	8	182	796	20	47	75
Paper questionnaire	8	3%	65	171	440	2	44	79
Telephone interview	1	1%	29	29	29	n.d.	n.d.	n.d.
Not available	79	33%	n.d.	n.d.	n.d.	n.d.	n.d.	n.d.
Total	234	100%	8	474	11,243	2	51	94

**Table 5:** IEQ categories considered.

Category	Number of Papers	Frequency (%) of Articles that Analyze this Category, Compared to those that Do Not	Main Variables Measured	Main Instruments Used for the Sampling/Measurement
Thermal comfort	134	85%	Temperature	HOBO and Tinytag sensors; Raspberry-Pi-based sensors; Kestral 4000 m; DT-172 logger; and HWM Ecosense temperature loggers
			Humidity	HOBO and Tinytag sensors; Raspberry-Pi-based sensors; Kestral 4000 m; and DT-172 logger
			Air pressure	HOBO and Tinytag sensors
			Air velocity	T-DCI-F900-S-O
Indoor air quality	100	64%	CO	HD21AB/HD21AB17
			Particulate matter, PM10 and PM2.5	Optical particle counters
			NO_2_	Passive Difram100 Rapid air monitor
			Total volatile organic compounds (TVOC)	RadielloTM Cartridge Adsorbents; 98,519
			Formaldehyde	Passive devices
			CO_2_	Raspberry-Pi-based sensors; 98,123 J; HD21AB/HD21AB17; Vaisala CO_2_ sensor
Lighting	112	71%	Lighting	TM-203 Datalogging; Digital Light Meter and Lutron-YK2005LX; illuminance sensors
			Glare	Camera-based imaging luminance photometer
			Views from windows	Two-dimensional color analyzer
Acoustic environment	97	62%	Noise	Sound level meter and tapping machine. Solo 1092 01dB-METRAVIB

**Table 6:** Number of studies per category.

Legend	Parameter/s	Number of Papers
A = lighting	A	10
B = acoustic environment	B	3
C = thermal comfort	C	17
D = indoor air quality	D	3
	AB	4
	BC	7
	CD	16
	AD	1
	ABC	12
	BCD	4
	ACD	5
	ABD	3
	ABCD	68
